# [Corrigendum] Ginsenoside Rg1 inhibits inflammatory responses via modulation of the nuclear factor-κB pathway and inhibition of inflammasome activation in alcoholic hepatitis

**DOI:** 10.3892/ijmm.2024.5407

**Published:** 2024-07-31

**Authors:** Jiajun Li, Cheng Yang, Shu Zhang, Shu Liu, Luole Zhao, Huan Luo, Yatang Chen, Wenxiang Huang

Int J Mol Med 41: 899-907, 2018; DOI: 10.3892/ijmm.2017.3297

Following the publication of the above article, the authors drew to the attention of the Editorial Office that, after having reviewed all the figures and the data of their drawing software, they discovered that the pictures in the 'Control' and 'DEX' groups of Fig. 4D on p. 904 had been incorrectly imported into Fig. 6 on p. 905 when assembling this figure, effectively replacing the original and correctly placed images in Fig. 6D and E.

The original (and correct) version of Fig. 6 is shown on the next page. All the authors agree with the publication of this Corrigendum, and express their gratitude to the Editor of *International Journal of Molecular Medicine* for allowing them the opportunity to publish this; furthermore, they apologize to the readership of the Journal for any inconvenience caused.

## Figures and Tables

**Figure 6:**
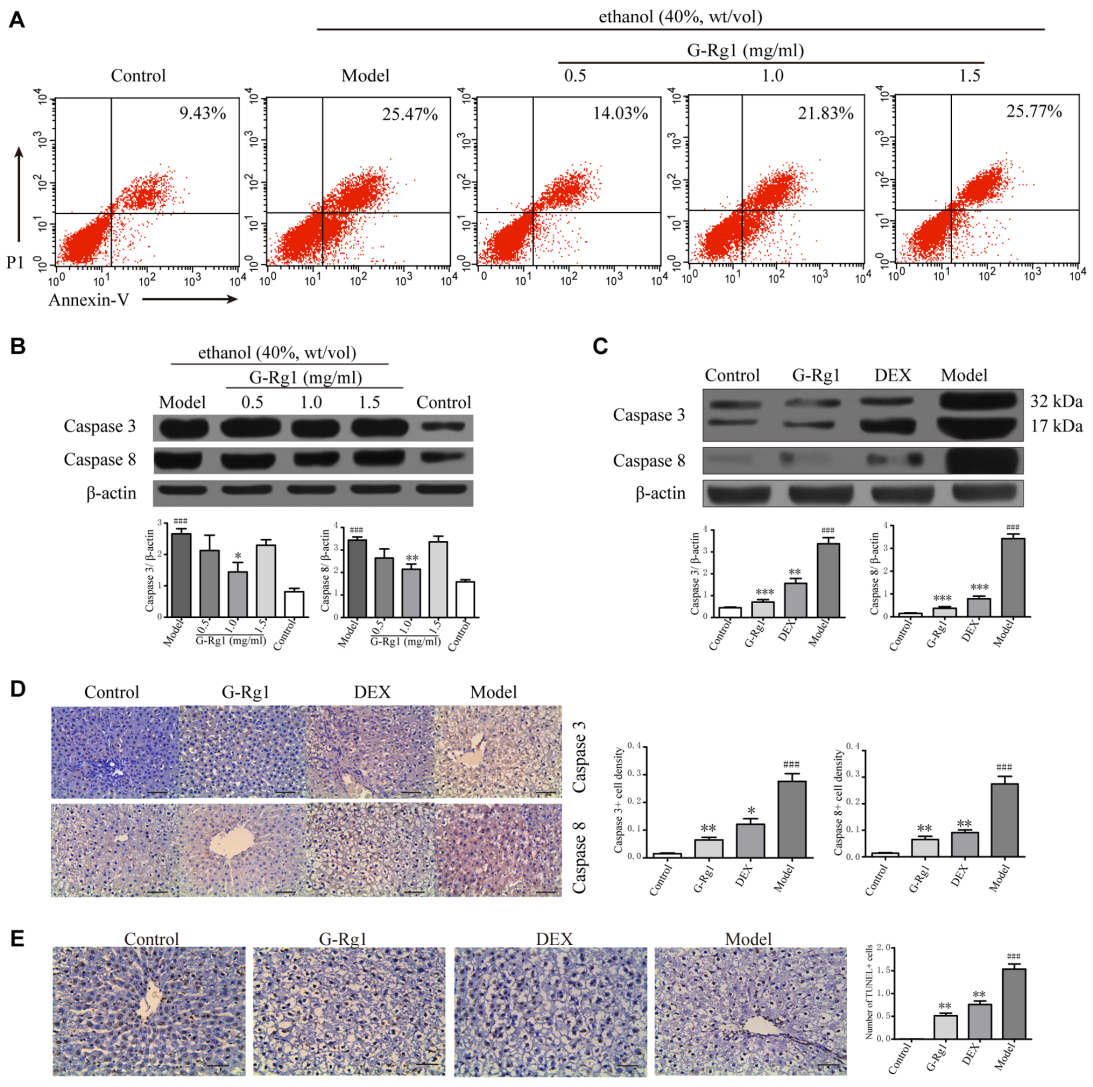
G-Rg1 inhibits hepatocellular apoptosis. L-O2 cells were exposed to ethanol and were treated with or without G-Rg1. (A) Flow cytometric analysis was used to detect apoptosis of L-O2 cells following alcohol exposure. (B) Western blot analysis of caspase-3 and -8 expression in L-O2 cells. (C-E) Rats with alcoholic hepatitis were administered G-Rg1 or DEX treatment, and caspase-3 and -8 expression was detected in liver tissues by (C) western blotting and (D) immunohistochemistry. (E) Apoptosis of hepatocytes was determined by terminal deoxynucleotidyltransferase-mediated dUTP nick end labeling assay. Scale bar, 50 *μ*m. Data are presented as mean ± standard error of the mean. ^###^P<0.001 vs. the control/saline group; ^*^P<0.05, ^**^P<0.01 and ^***^P<0.001 vs. the model/ethanol group. Results are from three independent experiments. DEX, dexamethasone; G-Rg1, ginsenoside Rg1.

